# Effectiveness of Digital Health Intervention in Enhancing Medication Adherence Among Arthritis Patients: A Systematic Review of Randomized Controlled Trials

**DOI:** 10.3126/nje.v14i4.77347

**Published:** 2025-09-01

**Authors:** Kshitija Gajadhur, Riya Patil, Arasi Pearia Anadachee, Abhishek Kashyap, Indrajit Banerjee

**Affiliations:** 1,2,3,5 Sir Seewoosagur Ramgoolam Medical College, Belle Rive, Mauritius; 4 Balrampur Hospital, Lucknow, Uttar Pradesh, India

**Keywords:** Arthritis, Digital health, Gout, Medication Adherence, Telemedicine

## Abstract

**Background:**

Arthritis affects millions of people worldwide; however, its mismanagement remains a growing global challenge, resulting in reduced quality of life (QoL) and disability. Digital health (DH), including smartphones, could be the key to solving this problem. Specific evidence-based reviews on the use of DH in this context are lacking. This systematic review aimed to explore the effect of DH on medication adherence (MA) among arthritis patients, thus improving their QoL.

**Methods:**

A literature search was conducted on PubMed, Cochrane, TRIP, and Google Scholar using the keywords digital health, medication adherence, and arthritis. All randomized controlled trials (RCT) published between 2006 and 2024 were assessed for eligibility.

**Result:**

The literature search yielded 12,671 results with 882 duplicates. The review included 8 RCTs that addressed rheumatoid arthritis (n=5), gout (n=2), and juvenile idiopathic arthritis (n=1), with a total sample size of 2449 patients, among whom 66% were males. Digital interventions used include calls, texts, videos, and online counselling. Study outcomes were classified as positive (DH increased MA), no significant difference, and negative (DH failed to show an effect on MA) in 62.5%, 25%, and 12.5% of the studies, respectively.

**Conclusion:**

It is concluded that 62.5% of the RCTs showed significant contributions of digital health tools in arthritis management, mainly by enhancing medication adherence and thus improving patients' QoL. Owing to their availability, simplicity, and immediacy, digital health tools demonstrate their potential as reliable interventions for supporting arthritis patients.

## Introduction

Arthritis, defined as the inflammation of joints affects more than 350 million people globally and from 2019 to 2021, 53.2 million U.S. citizens were diagnosed with it [1-3]. Being among the leading causes of disability in the USA, it plays a role in the workforce demography, quality of life, and other aspects of the patient’s life [4]. During the year 2013, the likelihood of American arthritis patients working was 7.2% points lower than that of healthy individuals. This is due to chronic pain, joint stiffness, reduced mobility, increased disease severity, and psychological factors [5]. According to the 2013 Medical Expenditure Panel survey conducted in the U.S, it was observed that arthritis patients ($9,233) had the highest level of annual medical expenditures as compared to adults with at least 1 chronic condition, excluding arthritis ($6,272) and those with no chronic illnesses ($1,369). The medical costs comprised of 4 domains: ambulatory care, inpatient care, prescriptions and other care [5].

Approximately 100 types of arthritis have been identified which can be broadly classified into 2 categories: Osteoarthritis and Inflammatory arthritis [2,3]. Inflammatory arthritis, also referred to as Autoimmune arthritis, is so termed because one’s immune system attacks healthy joints and tissues which lead to the inflammation and damage of joints, and includes: rheumatoid arthritis, gout, ankylosing spondylitis, psoriatic arthritis, lupus, enteropathic arthritis and polymyalgia rheumatica, among others [1,2]. The most common form of arthritis in the UK is osteoarthritis [1] and rheumatoid arthritis is the most common type of inflammatory arthritis [2]. commonly manifests as swelling, pain, stiffness of joints with decreased range of motions [6], and mental health illnesses such as depression and anxiety [7,8]. Most types of arthritis are not curable and can only be managed by medication to decrease disease activity. However, it has been observed that medication non-compliance is common among arthritis patients. Some of the reasons for this behaviour include forgetfulness, inadequate understanding of the disease, not knowing how to deal with adverse effects of the drugs, beliefs of the person, cost of medications, and the complex nature of the treatment, among others [9, 10].

Many of the manifestations and complications associated with arthritis could be prevented by effective outpatient management. Technologically driven alternatives such as digital health tools have been identified as promising to increase medication adherence, thus improving patient outcomes. Digital health can be described as using digital technologies in the medical field to manage illnesses, including wearable devices, electronic health records, and telemedicine [11]. There are multiple ways digital health can support medication compliance, it comprises digital interventions such as reminder texts to take medicines, electronic pill monitoring, educational videos to explain the pathogenesis of the disease and how to manage it, telephone or web-based counselling, gaming application, and so on.

The lifelong management of the disease, the financial burden of managing the condition, the increased costs and side effects due to non-adherence to medication highlight the need to find a suitable solution to increase medication adherence, which is now possible with the advent of accessible digital health tools, hence explaining the relevance of the study. Therefore, this systematic review aimed to evaluate how digital health interventions can improve medication adherence among arthritis patients and hence improve the patient’s quality of life.

## Methodology

The Preferred Reporting Items for Systematic Reviews and Meta-Analyses (PRISMA) 2020 guidelines were used to conduct this systematic review [12].

### Search strategy and selection criteria

A comprehensive literature review was performed using the following databases- PubMed, Cochrane Central Register of Controlled Trials (CENTRAL), Turning Research into Practice (TRIP), and Google Scholar. The Medical Subject headings (MeSH) terms and boolean operators used were Digital health OR Electronic health record OR Medical informatics OR Medical informatics applications OR Wearable Electronic Devices OR Telemedicine AND Medication adherence OR Patient compliance OR Treatment adherence and compliance AND Arthritis. [Table 1]

### Inclusion criteria

Completed randomized controlled trials (RCT) on digital interventions targeting medical adherence in arthritis patients were screened thoroughly. Full-text RCTs published in English between January 2006 and December 2024 were identified and incorporated into the systematic review.

### Exclusion criteria

Trials were excluded from this systematic review based on the lack of correlation between medication adherence and digital health among arthritis patients. Non-randomized clinical trials, cohort studies, case-control studies, cross-sectional studies, abstracts, case studies, reports, editorials, viewpoints, case series, and letters to the editor or correspondence manuscripts were also rejected.

### Study identification and selection

Four authors independently conducted blinded screening using the Rayyan platform based on titles and abstracts, adhering to the predefined selection criteria [13]. The full texts of eligible articles were examined for final selection, and a backward citation chase was carried out. Any newly identified eligible articles were screened and included in the final list of selected RCTs. [Table 2]

### Data extraction

The final list of selected RCTs was assessed. A data extraction table was made using Google Sheets that included the country of the study, digital intervention used, eligibility criteria of participants, sample size, size of intervention group, size of control group, measurement of medical adherence, study outcome or results, and limitations of the study.

### Risk of bias assessment

The Cochrane risk of bias tool for randomized trials (RoB 2) [14] was used for risk of bias assessment, and the compiled data were transferred to the Robvis visualization tool [15] for generating traffic light plots and weighted bar plots.

## Results

**Figure 1:**
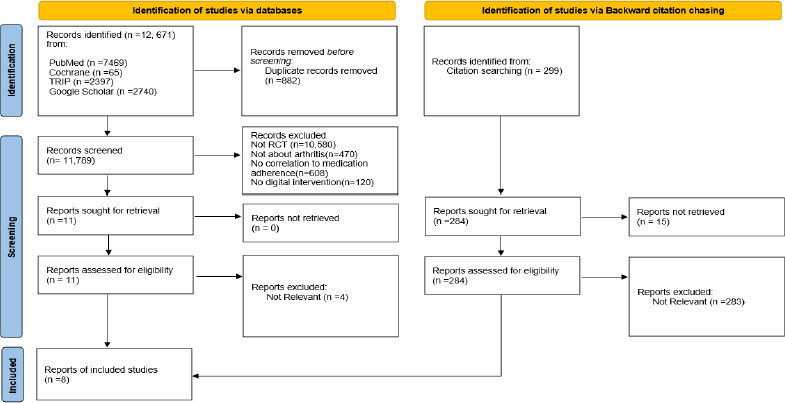
PRISMA flow diagram for new systematic reviews which included searches of databases and other sources [12]

A total of 12,671 articles were identified in the initial literature review, of which 882 were duplicates, leaving 11,789 unique articles for further screening. Articles were eliminated based on their inability to meet the criteria of being an RCT, an arthritis population, correlation to medication adherence, or digital intervention. Consequently, assessment of 11 full-text articles were carried out based on the predefined selection criteria, of which 7 RCTs were deemed eligible. Backward citation chasing led to the identification of 1 more eligible article. Therefore, 8 RCTs were thoroughly evaluated for the impact of digital intervention on medication adherence among arthritis patients. [ Figure 1]

**Figure 2:**
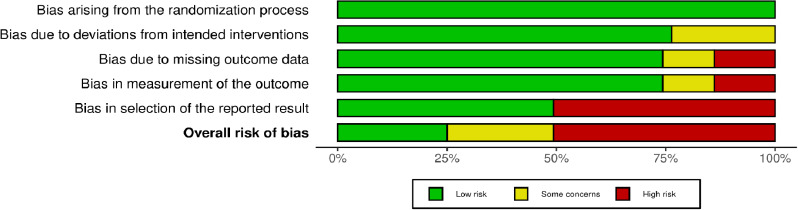
Weighted bar plot

**Figure 3:**
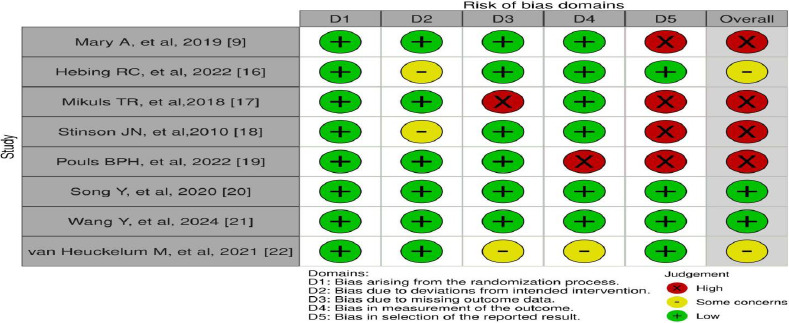
Traffic light plot

According to the traffic light plot (Figure 3), 4 articles were high risk, followed by 2 of some concerns and 2 of low risk. As displayed by the weighted bar plot (Figure 2) and traffic light plot (Figure 3), the domain displaying the most significant amount of bias was D5(Bias in the selection of reported result) followed by D4 (Bias in measurement of the outcome), D3(Bias due to missing outcome data) and D2 (Bias due to deviation from intended interventions). The least bias was seen in D1(Bias arising from the randomization process).

Some studies assessed additional demographic data, including medical insurance by 2 RCTs, degree of education by 4 RCTs, caregiver or parent role by 1 RCT, and the effect of concomitant medication use by 1 RCT. Disease duration, comorbidities, marital status, and occupation status were assessed by 4 RCTs and 2 RCTs.

Five RCTs assessed medication adherence as the primary outcome, while the remaining 3 RCTs assessed it as a secondary outcome. Additional outcomes assessed were remission time, disease activity, serum uric acid level, and disease comprehension.

Table 3 depicts the Author, Country of study, Digital intervention used, Targeted population, Eligibility criteria of participants, sample Size, size of intervention group, size of control group, and Ancillary staff associated with care.

Table 4 depicts the medication used, measurement of medication adherence, study outcome/ results, analysis grouping, [95% CI], and various limitations of the selected RCTs.

## Discussion

Previous studies by Peng Y et al. (2020) [23] and Badawy SM et al. (2017) [24] have revealed the favorable effect of mobile phone applications on medication adherence in several chronic illnesses. However, dedicated reviews exploring the effectiveness of these measures among arthritis patients are limited. In this systematic review, 8 RCTs analyzing multiple digital health interventions among rheumatoid arthritis and gout patients were evaluated. Improvement in medication compliance with the intervention was noted in 5 trials, emphasizing the importance of telemedicine in arthritis medication management.

Hebing RC et al. (2022) conducted an RCT among 206 rheumatoid arthritis patients to study the effectiveness of electronic drug monitoring on biological disease-modifying antirheumatic drug (bDMARD) adherence. The intervention group patients received a needle disposal container with a medication event monitoring system cap and regular motivational interviewing (MIT)-based feedback by pharmacists [16]. The electronic drug monitoring feedback increased medication adherence as measured by the medication possession ratio (MPR) at the end of 12 months. bDMARD naive patients benefited the most as they achieved faster improvements in disease activity. The MIT-based feedback was a powerful aspect of this study and has impacted adherence the most. However, patients were aware of the intervention, which may have affected their behavior and led to the high adherence rates.

Mary A. et al. (2019) performed an RCT using mobile phone text message reminders to increase methotrexate adherence in rheumatoid arthritis patients. The intervention positively impacted medication adherence as measured by the change over time in the Compliance Questionnaire Rheumatology (CQR-19) score, MPR, and Girerd score [9]. Furthermore, patient satisfaction with using this intervention was high, and the improvement in adherence was found to be independent of baseline patient characteristics.

Pharmacist or nurse-led tailored telephone interventions were also beneficial in increasing medication adherence. Mikuls TR et al. (2018) conducted an RCT among gout patients, with the intervention group receiving regular pharmacist-delivered telephone calls [17]. Intervention group patients achieved high allopurinol adherence rates and lower serum urate levels. A similar study by Song Y et al. (2020) confirmed the positive influence of a 12-week nurse-led telephone-delivered educational intervention among rheumatoid arthritis patients. Study group patients had notably higher treatment compliance than the standard group at the end of the intervention [20].

The effectiveness of mobile phone applications in gout medication management was validated by the findings of an RCT conducted by Wang Y et al. (2024), which used a gout self-management app to increase disease knowledge and treatment compliance. The Gout Intelligent Management app provided continuous patient care, collected health records, and provided health education and interactive support. Favorable results were obtained in the Chinese Compliance Questionnaire Rheumatology (CCQR) score, serum uric acid levels, and Gout Knowledge Questionnaire (GKQ) score [21].

Despite most RCTs reporting positive outcomes, a pilot nonblinded RCT conducted by Pouls BPH et al. (2022) showed contradictory findings. The intervention arm of the study, consisting of 113 rheumatoid arthritis patients, received usual face-to-face consultations along with instructions for downloading and playing a serious puzzle game app aiming to improve medication adherence. Although user engagement with the app was high, the gaming intervention failed to improve medication adherence. The negative result can be attributed to the non-integration of the app in the patient care pathway and behavioral task effectiveness. Despite disagreeable findings, the app showed promise and capacity for further improvement and modifications [19].

Two other trials included in this review showed no significant impact of digital health technologies on medication adherence. Stinson JN et al. (2010) conducted a study focusing on Juvenile Idiopathic Arthritis (JIA) patients using a website and telephone-delivered multicomponent health coach-guided intervention to increase treatment compliance. Intervention group patients spent 20-30 minutes per week completing a module on the Internet and receiving weekly telephone calls. Adherence was measured using the JIA-specific Child Adherence Report Questionnaire (CARQ) and Parent Adherence Report Questionnaire (PARQ). This study also considered parents' role in promoting and encouraging treatment compliance. Although medication adherence did not improve, favorable effects were seen on the pain intensity and disease-specific knowledge. Limitations that may have resulted in such an outcome include a small sample size and lack of an appropriate control group [18].

Similar findings were reported from a trial performed by Van Heuckelum M et al. (2021), in which the use of electronic monitoring feedback in rheumatoid arthritis patients failed to show any significant improvement in medication adherence. This study, however, had multiple flaws, including selection bias, technical difficulties, and high attrition rates, that may have compromised the study outcome [22].

This systematic review has a few study limitations. It only includes trials on rheumatoid arthritis, gout, and Juvenile Idiopathic Arthritis patients since limited RCTs are available on the effect of mobile health interventions on other types of arthritis and their treatment protocols. Other shortcomings include small sample size, lack of generalizability, missing outcome data, and deviations from the intended intervention in some of the included studies.

A significant factor leading to medication non-compliance among arthritis patients is the high cost of medications, as reflected in the worsening clinical outcomes in economically underprivileged patient groups [25]. Barring two studies conducted in China by Song Y et al. (2020) [20] and Wang Y et al. (2024) [21] that considered the medical insurance details of the patients, none of the other trials took cost-related medication non-adherence into account. Future studies incorporating this aspect of treatment compliance are required to develop strategies and interventions to improve medication adherence, especially among the lower socioeconomic class of patients.

In recent years, several attempts have been made to develop new health technologies for improving treatment adherence among arthritis patients. Feasibility studies such as those conducted by Lim S et al. (2023) [26] and Butler S et al. (2024) [27] have identified the effectiveness and usability of such recently developed digital health tools. This systematic review re-enforces their findings and establishes the massive potential of mobile and web-based health tools in becoming vital for treatment compliance in this fast-advancing digital world. Although telemedicine interventions offer great promise, additional trials on larger study populations suffering from other forms of arthritis and technological improvements are required to develop user-friendly and easily accessible technologies.

## Conclusion

Arthritis is a chronic debilitating illness requiring lifelong treatment and symptom management. Electronic drug monitoring feedback, mobile phone text messages, telephone calls, and other digital health technologies can improve medication adherence in arthritis patients through regular monitoring and motivational counselling. Improved treatment compliance would drastically modify disease activity and quality of life. However, the long-term effectiveness of these patient-centered digital interventions remains unexplored and requires further study.

## Figures and Tables

**Table 1:** Search strategy

Database	Keywords and Boolean operators	Filters used	Results obtained
**PubMed**	Digital health OR Electronic health record OR Medical informatics OR Medical informatics applications OR Wearable Electronic Devices OR Telemedicine OR Patient compliance OR Treatment adherence and compliance AND Arthritis OR Arthritis, infectious OR Psoriatic arthritis OR Rheumatoid arthritis OR Gout OR Chondrocalcinosis OR Osteoarthritis OR Periarthritis OR Sacroiliitis OR Spondyloarthritis AND Medication adherence	2006-2024 English Human RCT	7,469
**Cochrane Central Register of Controlled Trials (CENTRAL)**	Digital health OR Electronic health record OR Medical informatics OR Medical informatics applications OR Wearable Electronic Devices OR Telemedicine OR Patient compliance OR Treatment adherence and compliance AND Arthritis OR Arthritis, infectious OR Psoriatic arthritis OR Rheumatoid arthritis OR Gout OR Chondrocalcinosis OR Osteoarthritis OR Periarthritis OR Sacroiliitis OR Spondyloarthritis AND Medication adherence	2006-2024 English Trials	65
**Turning research into Practice (TRIP)**	Digital health OR Electronic health record OR Medical informatics OR Medical informatics applications OR Wearable Electronic Devices OR Telemedicine OR Patient compliance OR Treatment adherence and compliance AND Arthritis OR Arthritis, infectious OR Psoriatic arthritis OR Rheumatoid arthritis OR Gout OR Chondrocalcinosis OR Osteoarthritis OR Periarthritis OR Sacroiliitis OR Spondyloarthritis AND Medication adherence	2006-2024 English RCT	2,397
**Google Scholar**	Digital health OR Electronic health record OR Medical informatics OR Medical informatics applications OR Wearable Electronic Devices OR Telemedicine OR Patient compliance OR Treatment adherence and compliance AND Arthritis OR Arthritis, infectious OR Psoriatic arthritis OR Rheumatoid arthritis OR Gout OR Chondrocalcinosis OR Osteoarthritis OR Periarthritis OR Sacroiliitis OR Spondyloarthritis AND Medication adherence	2006-2024 English	2,740
		**Total**	12,671

**Table 2:** Predefined selection criteria

Category	Inclusion criteria	Exclusion criteria
**Study Design**	Randomized controlled trials (RCT)	Non-randomized clinical trials, cohort studies, case-control studies, cross-sectional studies, abstracts, case studies, reports, editorials, viewpoints, case series, and letters to the editor/correspondence manuscripts
**Population**	Arthritis patients – Arthritis, infectious arthritis, Psoriatic arthritis, Rheumatoid arthritis, Gout, Chondrocalcinosis, Osteoarthritis, Periarthritis, Sacroiliitis, Spondyloarthritis, Enteropathic arthritis, Polymyalgia rheumatica	Any other population apart from arthritis patients
**Intervention**	Digital intervention – Digital health, electronic health record, medical informatics, medical informatics applications, mHealth, wearable electronic devices, telemedicine, medication monitoring device	Absence of digital intervention
**Comparison**	Control groups	
**Outcome assessed**	Medication adherence	Absence of medication adherence assessment
**Species**	Human	Animal
**Language**	English	Any other language apart from English
**Time range**	2006-2024	Any year outside of the range 2006-2024
**Free full text**	Available	Not available

**Table 3:** Author, Country of study, Digital intervention used, Targeted population, Eligibility criteria of participants, Sample Size, Size of intervention group, Size of control group, Ancillary staff associated with care

Author	Country of study	Digital intervention used	Targeted population	Eligibility criteria of participants	Sample Size	Size of intervention group	Size of control group	Ancillary staff associated with care
**Mary A, et al, [9]**	France	Weekly text message reminder	RA	Adult patients Consultation in associated rheumatology department for RA Treated with methotrexate for at least 3 months Has a mobile phone	112	37	38	Pharmacist
**Hebing RC, et al, [16]**	Netherlands	-Special needle disposal container -Medication Event Monitoring System cap	RA	Adult patients RA diagnosis consistent with 2010 ACR classification Use of subcutaneous administered Bdmard	206	104	102	Pharmacist
**Mikuls TR, et al, [17]**	USA	Telephone calls	Gout	Adult English-speaking Gout diagnosis New allopurinol prescription	1551	769	782	Pharmacist
**Stinson JN, et al, [18]**	Canada	Telephone calls and website delivered treatment protocol	JIA	12 to 18 years of age JIA diagnosis Able to speak English or French Complete baseline online outcome measures	46	22	24	Trained coach (non-healthcare professional with an undergraduate degree in psychology)
**Pouls BPH, et al, [19]**	Netherlands	Gaming application	RA	Clinical RA diagnosis DMARD use Self-management of medication Has a smartphone/tablet with an iOS/Android software and a valid email address	229	113	116	Pharmacist
**Song Y, et al, [20]**	China	Telephone calls	RA	Confirmed RA diagnosis based on the 2010 ACR/EULAR classification criteria 18 years old or older Discharged from hospital Proficient in Chinese Willing to participate in study	92	46	46	Nurse
**Wang Y, et al, [21]**	China	mHealth app (Gout Intelligent Management app)	Gout	Adult patients Confirmed Gout diagnosis based on 2015 gout classification Aware of disease status Able to use Android smartphone Can understand questionnaire and intervention	120	60	60	Nurse
**van Heuckelum M, et al, [22]**	Netherlands	Medication Monitoring device	RA	Adult patients RA diagnosis of less than 1 year by rheumatologist Use of methotrexate Sufficient proficiency in Dutch language No significant cognitive limitation No need for help in taking medications Life expectancy of at least 12 months	93	47	46	Nurse

RCT- randomised controlled trial; RA- Rheumatoid arthritis; JIA-Juvenile Idiopathic Arthritis; ACR-American College of Rheumatology; EULAR- European Alliance of Associations for Rheumatology; DMARD- disease-modifying anti-rheumatic drug

**Table 4:** Medication used, Measurement of medication adherence, Study outcome/ Results, Analysis Grouping, Values [95% Confidence Intervals], Limitations

Author	Medication used	Measurement of medication adherence	Study outcome/ Results	Analysis Grouping	Values [95% Confidence Intervals]	Limitations
**Mary A, et al, [9]**	Methotrexate	-CQR -Girerd score -MPR	Positive	Overall	3.63 (1.26 to 10.49)	Medication adherence was not assessed before intervention Selection bias No MPR data collected before intervention Outcome measured by CQR questionnaire was subjective
**Hebing RC, et al, [16]**	Bdmard	-MPR -CQR	Positive	Medication possession ratio	0.036 (0.001 to 0.007)	Selection bias
				Baseline Disease Activity Score	1.68 (1.00 to 2.81)	
**Mikuls TR, et al, [17]**	Allopurinol	Pharmacy dispensing data	Positive	Medication Adherence	1.68 (1.30 to 2.17)	Selection bias Outcome was not properly measured as most of the intervention group patients never received dose increases and failed to achieve target SU goal
				Serum Urate Goal	2.37 (1.83 to 3.05)	
**Stinson JN, et al, [18]**	Routine therapy of individual	-CARQ -PARQ	No significant effect on medication adherence	Not available	Not available	Selection bias as attention control group may not have been a fair comparator Deviation from intended intervention
**Pouls BPH, et al, [19]**	DMARD	-CQR -Electronic medication monitoring device -Pill/Syringe count	Negative	Medication adherence	-8 (-22 to 6)	Selection bias The absence of integration of the game in the RA care pathway
**Song Y, et al, [20]**	Routine therapy of individual	-CCQR	Positive	Medication adherence	0.58 (0.12 to 1.03)	Medication adherence was not assessed before intervention All individuals considered were from one hospital
**Wang Y, et al, [21]**	Routine therapy of individual	-CCQR	Positive	Gout knowledge levels	1.300 (0.669 to 1.931)	Individuals considered were from one hospital Long term effect of intervention were not studied
				Treatment adherence at 12^th^ wk	1.667 (-3.283 to 6.617)	
				Treatment adherence at 24^th^ wk	6.287 (1.357 to 11.216)	
**van Heuckelum M, et al, [22]**	Methotrexate	-CQR	No significant effect on medication adherence	Not available	Not available	Selection bias Recruitment limit the generalizability of study

RCT- randomised controlled trial; RA- Rheumatoid arthritis; JIA-Juvenile Idiopathic Arthritis; ACR-American College of Rheumatology; EULAR- European Alliance of Associations for Rheumatology; DMARD- disease-modifying anti-rheumatic drug; bDMARD- biologic disease-modifying anti-rheumatic drug, bDMARD- biologic disease-modifying anti-rheumatic drug; DMARD- disease-modifying anti-rheumatic drug, MPR- Medication Possession Ratio; CQR- Child adherence Report Questionnaire; SU- Serum urate; CARQ- Child Adherence Report Questionnaire; PARQ- Parent Adherence Report Questionnaire; CCQR- Chinese Compliance Questionnaire Rheumatology
